# Supplementary figures and images for: Inferring tumor-specific cancer dependencies through integrating ex vivo drug response assays and drug-protein profiling
Source: PLoS Comput Biol. 2022 Aug 22;18(8):e1010438. doi: 10.1371/journal.pcbi.1010438 (PMC9436053; doi:10.1371/journal.pcbi.1010438)

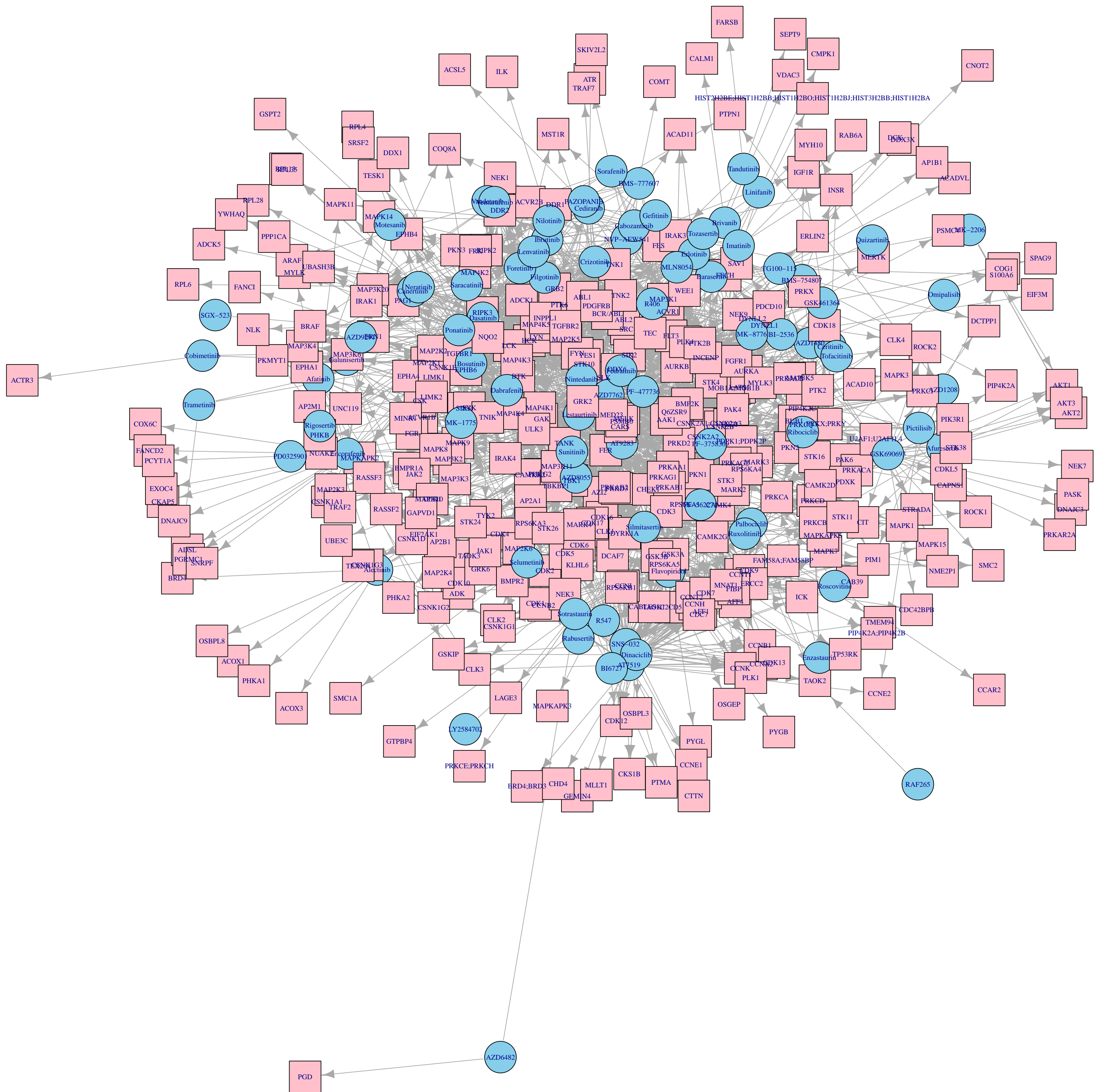

Supplement: S1 Fig — Only the high confidence drug-kinase pairs are included. Drugs are shown as blue circles and kinases are shown as red squares. No clear structure can be observed directly from the drug-kinase network. (PDF) [file pcbi.1010438.s001.pdf]

## GDSC drug screen

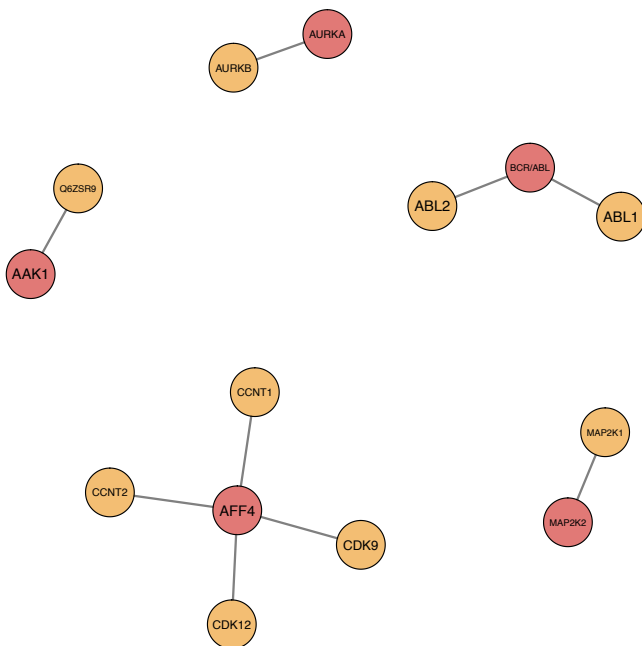

## BeatAML drug screen

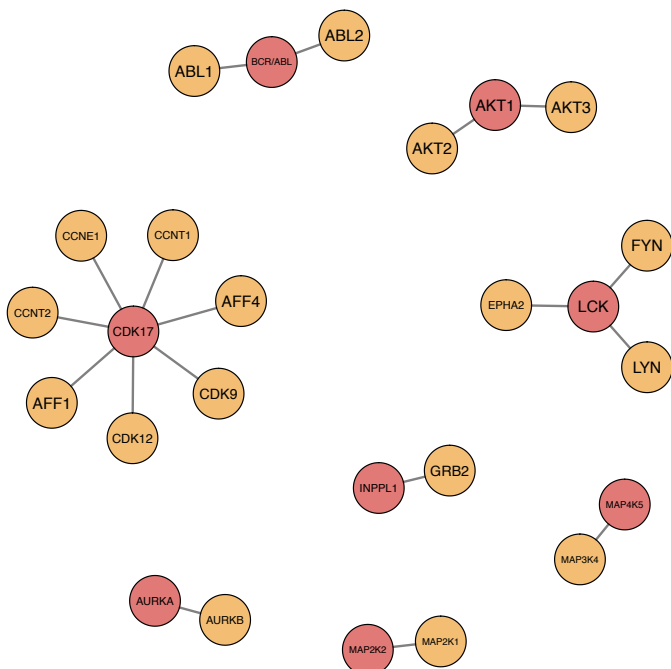

# EMBL2016 drug screen

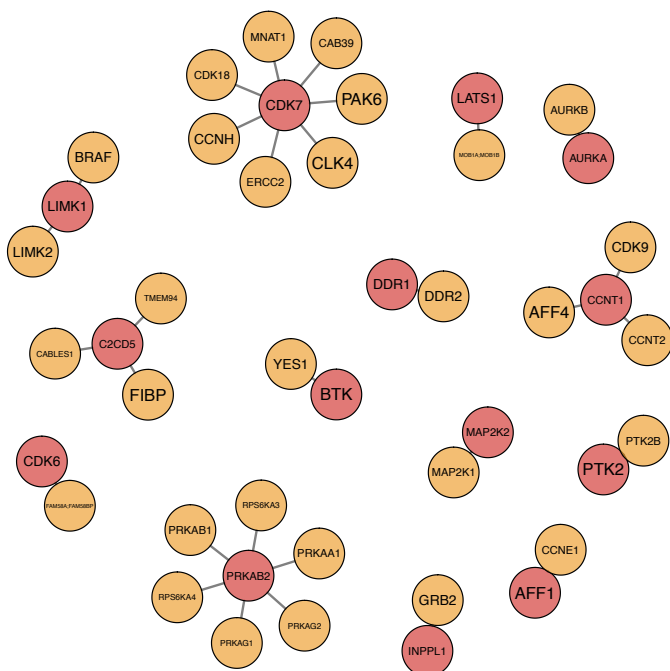

Supplement: S2 Fig — For each drug screen dataset, only the kinases that were selected by our protein dependence inference algorithm and share similar drug binding profiles with other kinases are shown here. The full list is provided in S1–S3 Tables. (PDF) [file pcbi.1010438.s002.pdf]

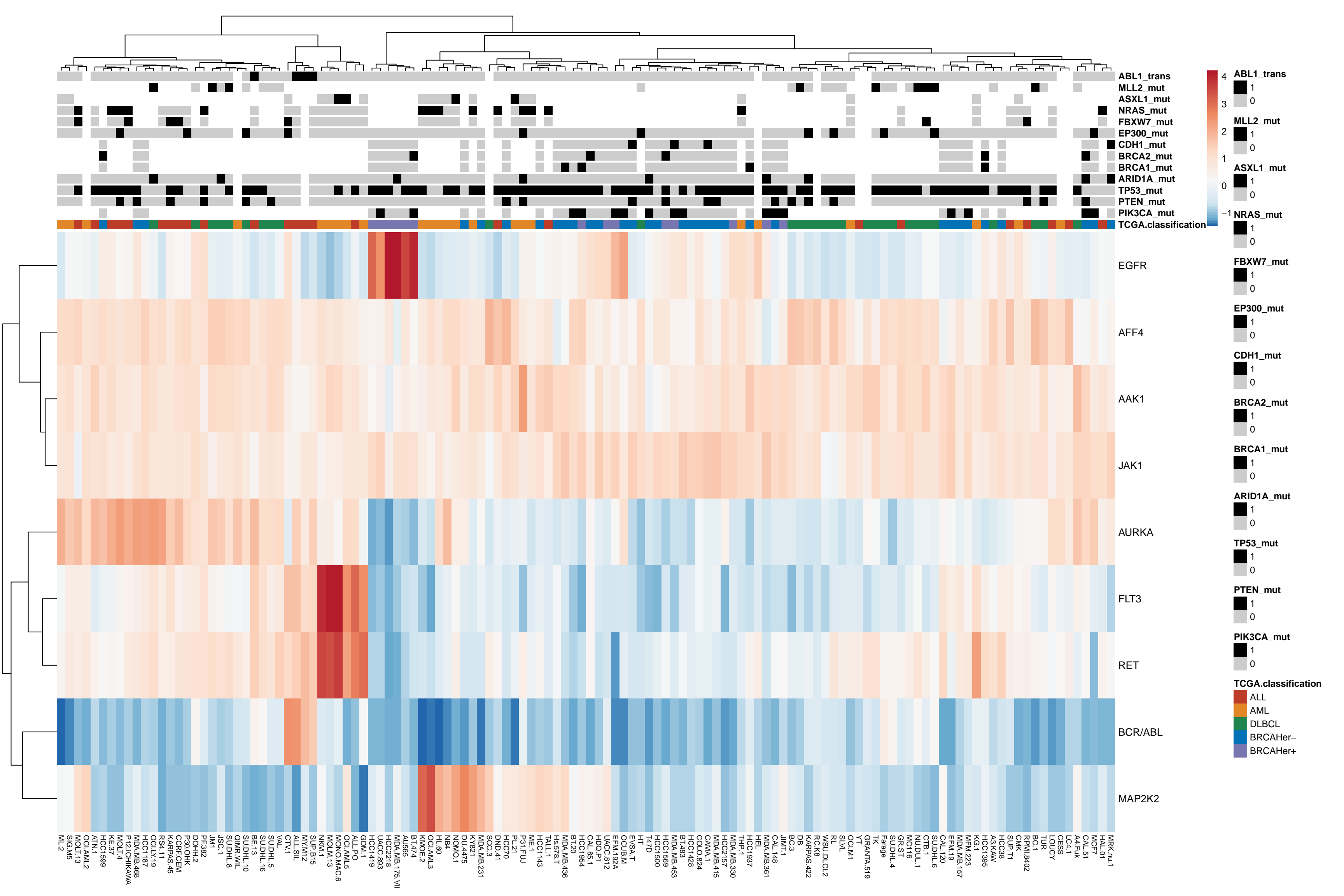

Supplement: S3 Fig — Red indicates high dependence value and blue indicates low dependence value. In the column annotations, cell lines with certain genomic variations are colored by black. Columns are ordered by hierarchical clustering based on Euclidean distance. (PDF) [file pcbi.1010438.s003.pdf]

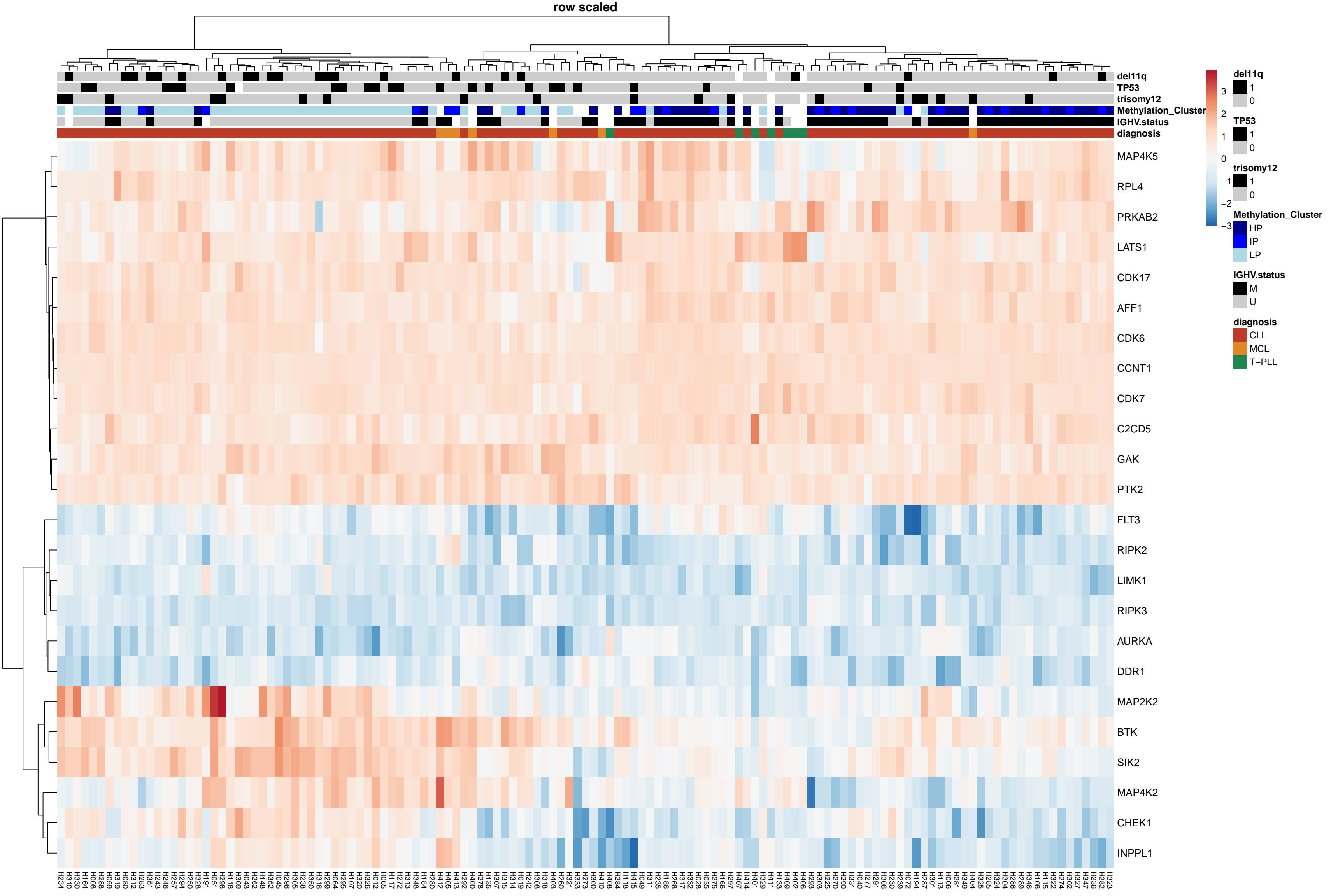

Supplement: S5 Fig — Red indicates high dependence value and blue indicates low dependence values. The column annotations of the heatmap indicate the disease types, genomic variations and epigenetic subtypes of the primary blood cancer samples. Columns are ordered by hierarchical clustering based on Euclidean distance. (PDF) [file pcbi.1010438.s005.pdf]

# BTK ~ diagnosis

Protein dependence

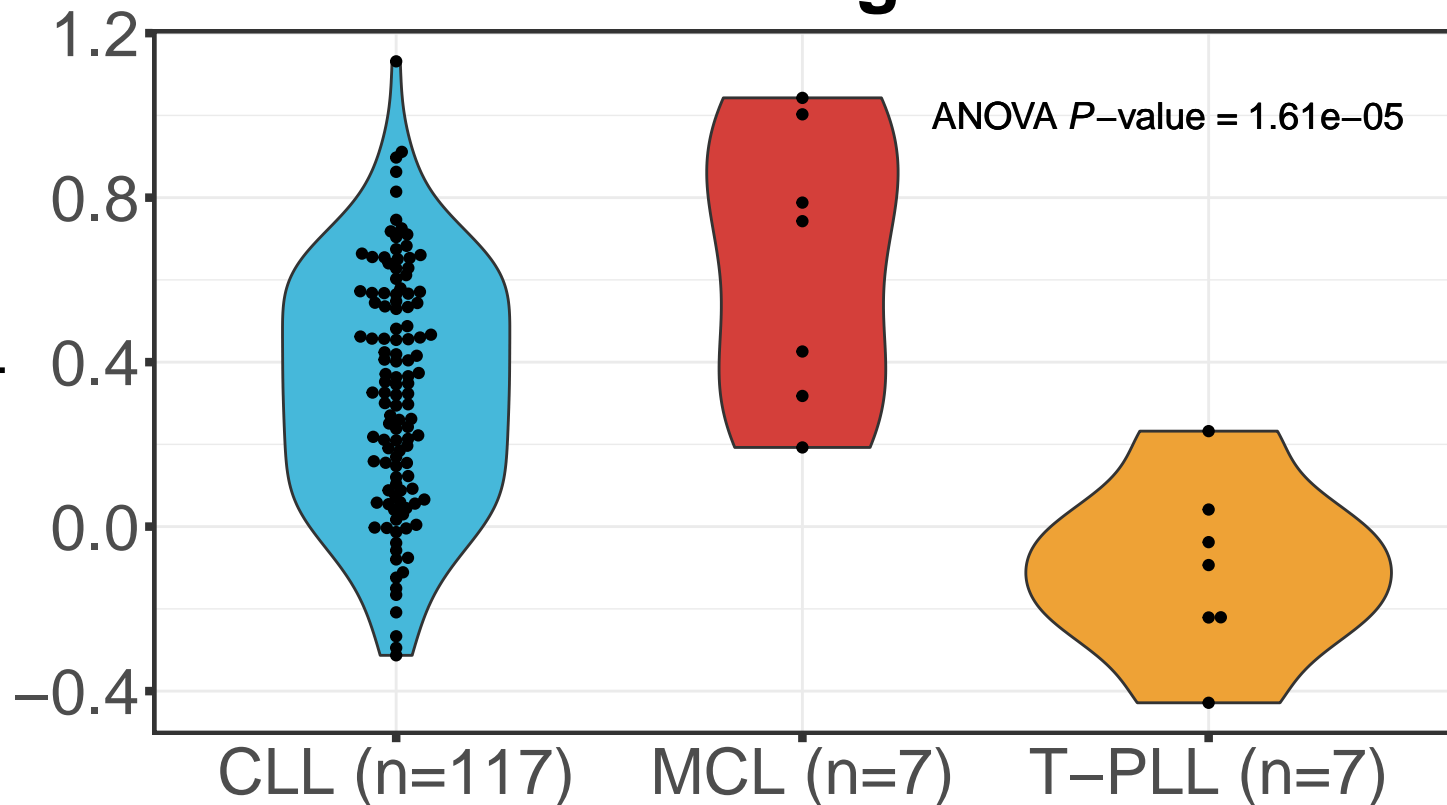

Supplement: S6 Fig — P value was calculated by one-way ANOVA test. To avoid potential bias due to the unbalanced sample size or unequal variance, nonparametric Kruskal-Wallis test was also performed and the resulting P value is 0.00019. (PDF) [file pcbi.1010438.s006.pdf]

# ICGC–CLL cohort (Hallmarks, 5% FDR)

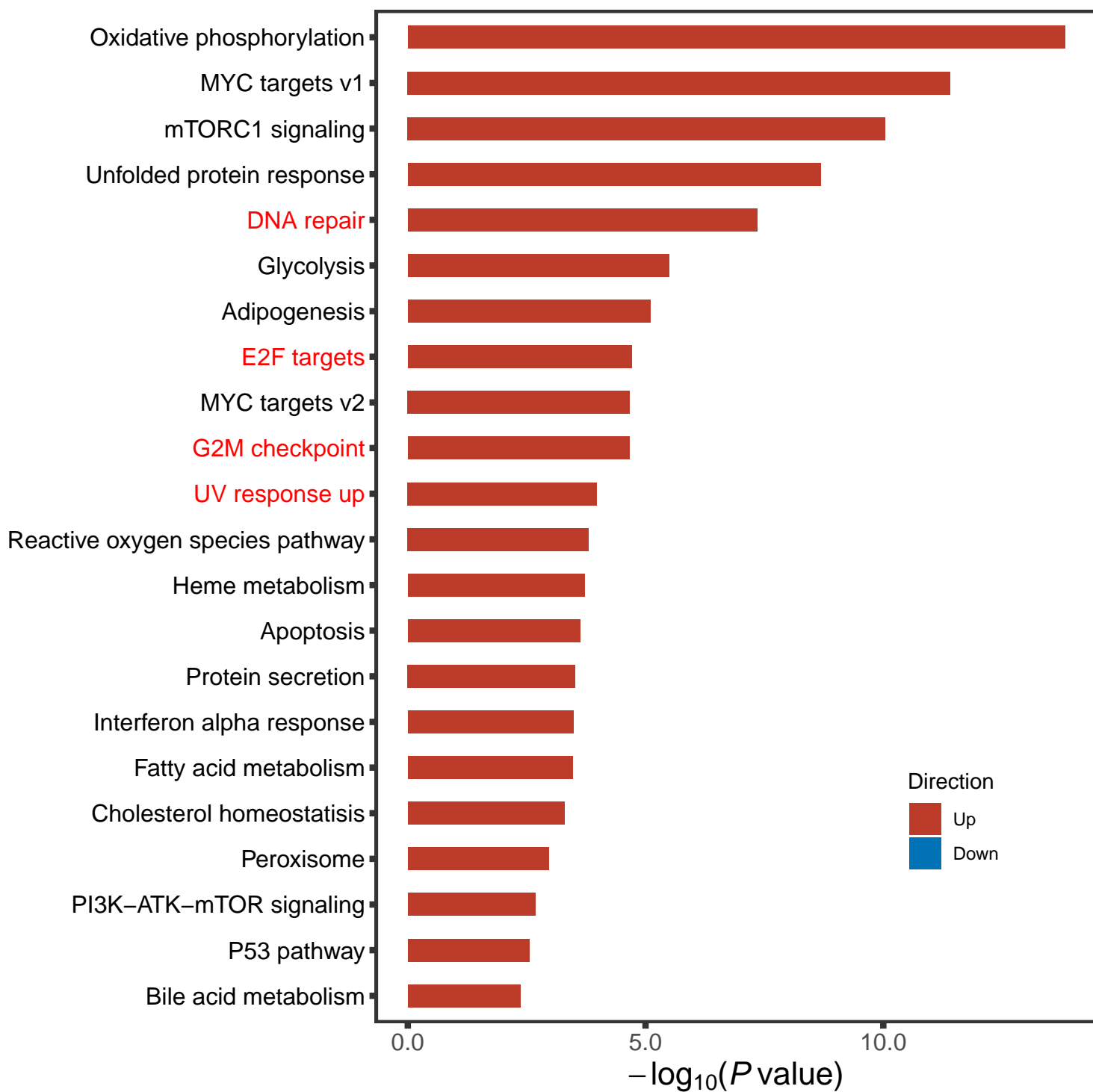

Supplement: S7 Fig — “Up” (colored by red”) indicates the gene sets enriched for the genes up-regulated in U-CLL samples compared to M-CLL samples. The enrichment analysis was performed by using CAMERA against the Cancer Hallmark gene sets from the Molecular Signature Database (MSigDB). Only the gene sets passed 5% FDR are shown. The gene sets that have been shown to be related to CHEK1 functions are colored in red. (PDF) [file pcbi.1010438.s007.pdf]

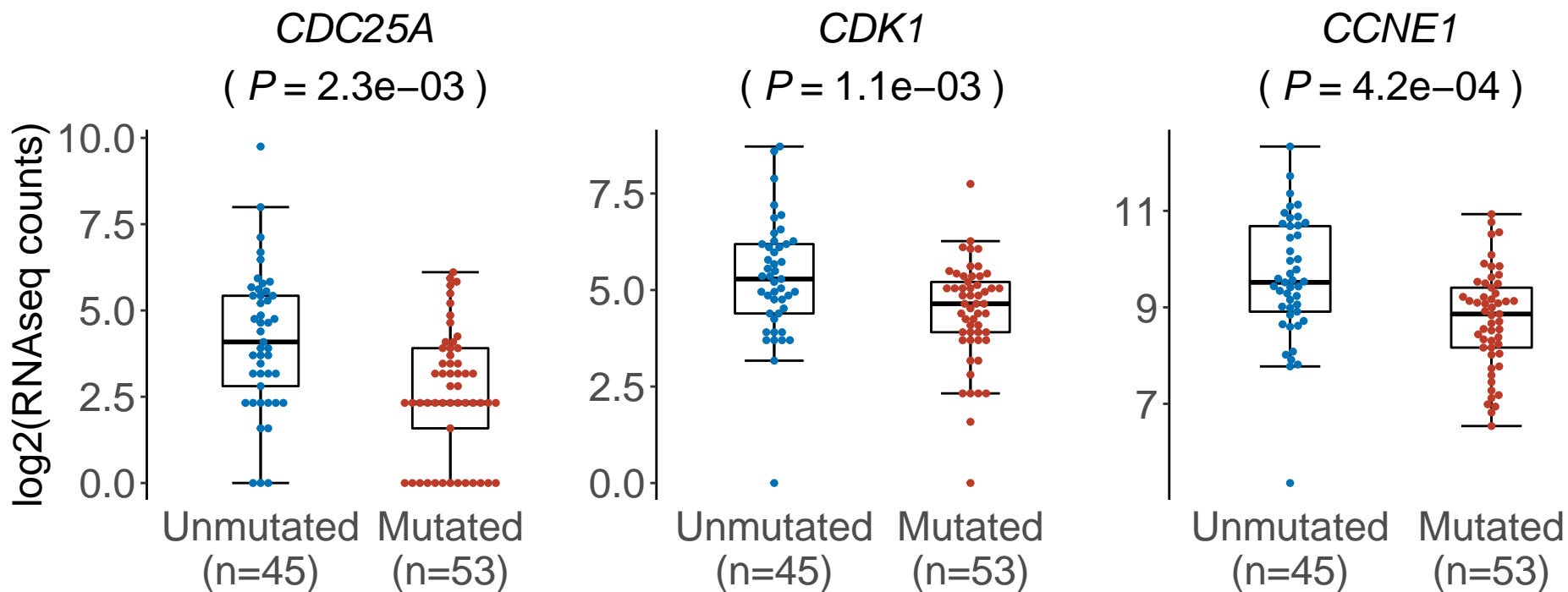

Supplement: S8 Fig — P values were calculated by DESeq2. (PDF) [file pcbi.1010438.s008.pdf]

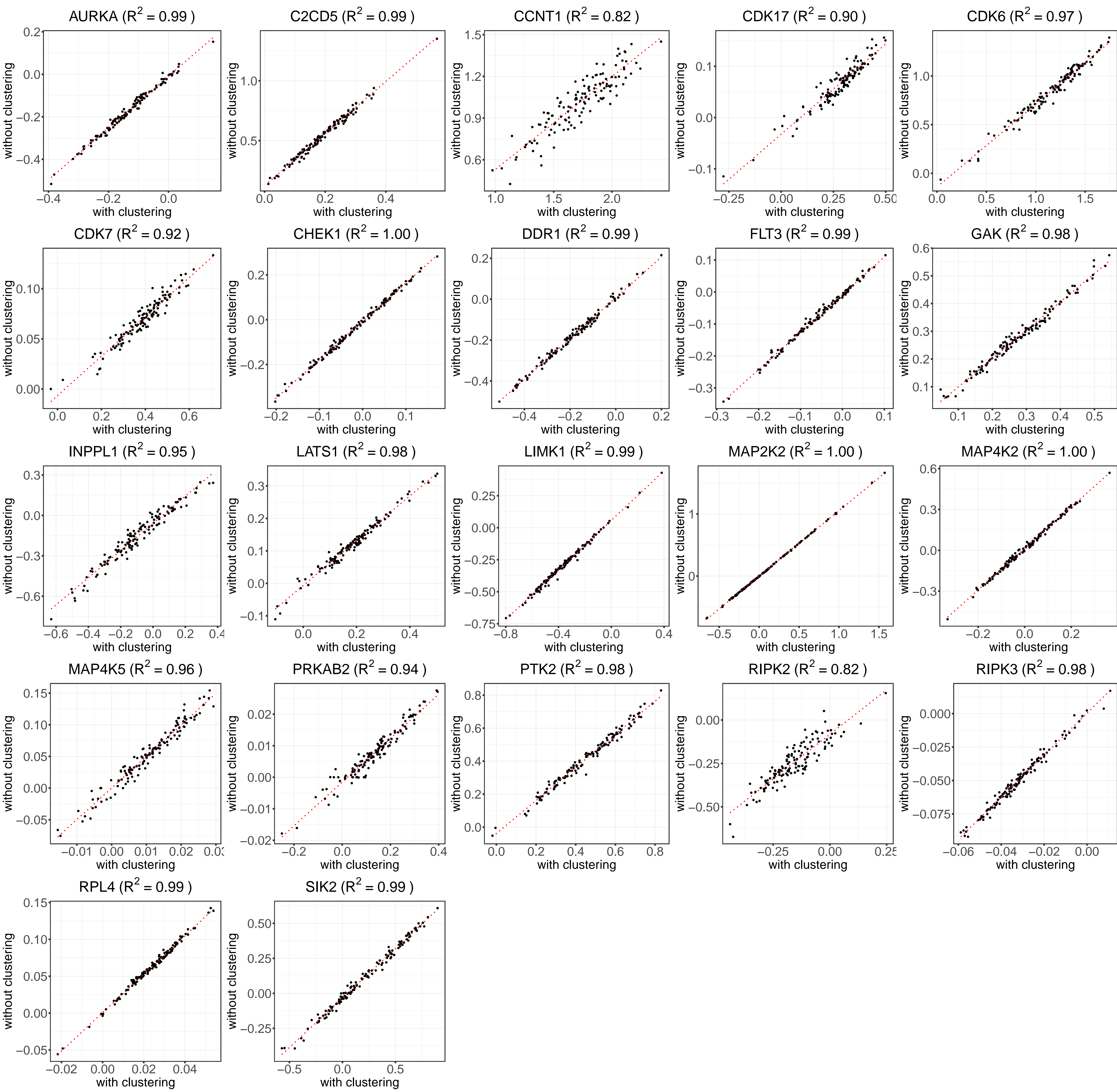

Supplement: S9 Fig — (PDF) [file pcbi.1010438.s009.pdf]

$$R^2 = 0.91$$

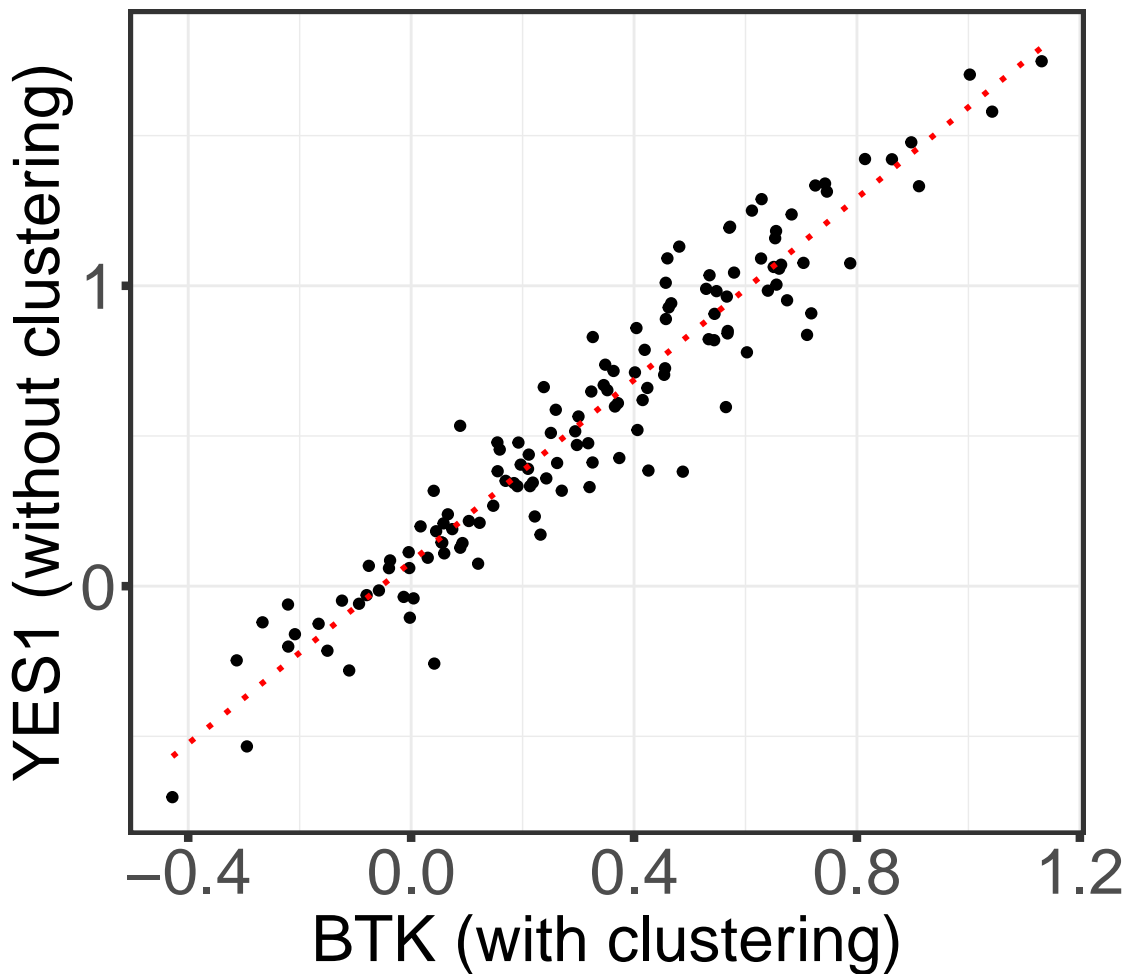

Supplement: S10 Fig — (PDF) [file pcbi.1010438.s010.pdf]

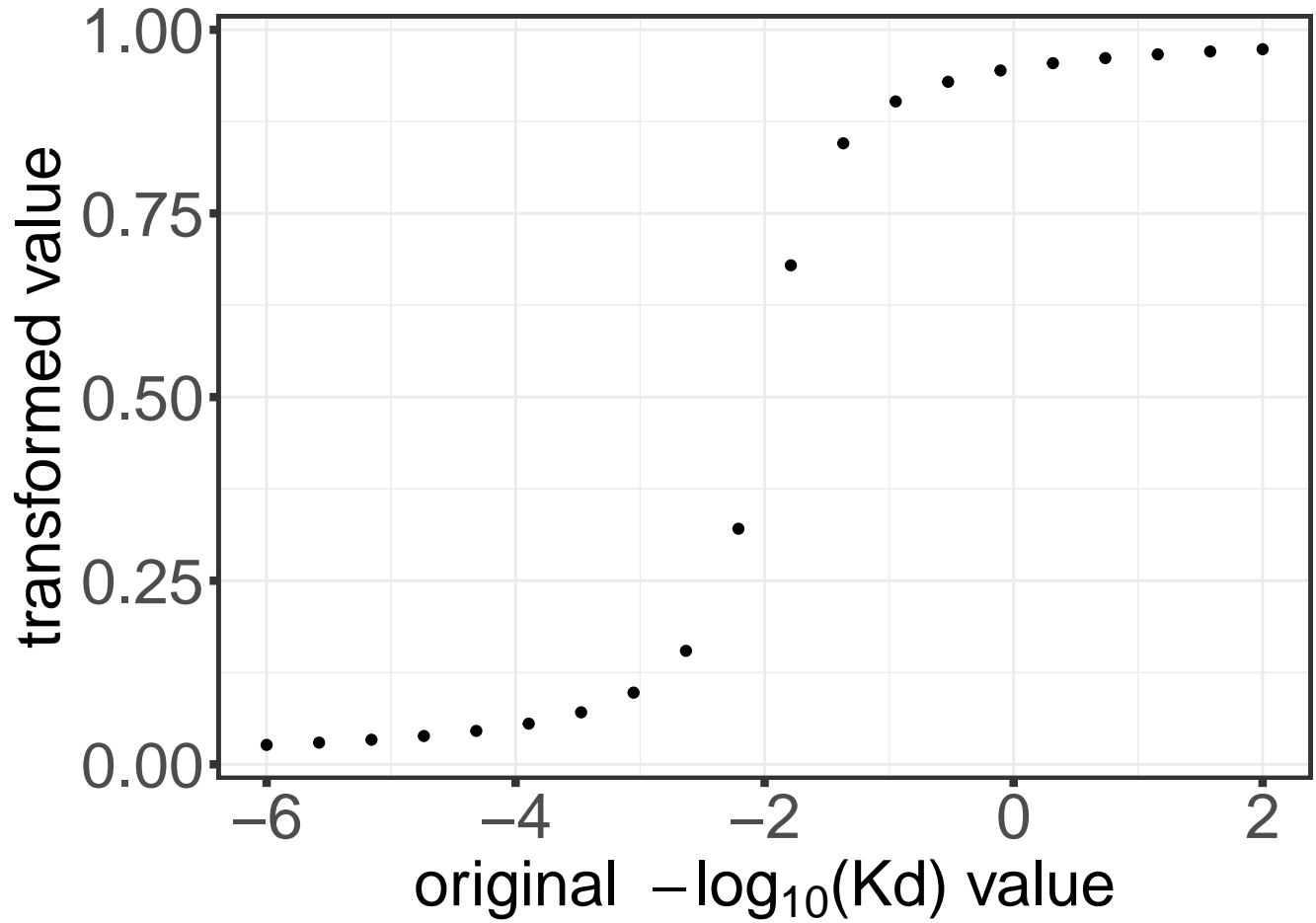

Supplement: S11 Fig — (PDF) [file pcbi.1010438.s011.pdf]
