# Supplementary material for: Inferring tumor-specific cancer dependencies through integrating ex vivo drug response assays and drug-protein profiling
Source: PLoS Comput Biol. 2022 Aug 22;18(8):e1010438. doi: 10.1371/journal.pcbi.1010438 (PMC9436053; doi:10.1371/journal.pcbi.1010438)
Supplement: S4 Fig — P values are from two-sided Student’s t-test with equal variance. (PDF) [file pcbi.1010438.s004.pdf]

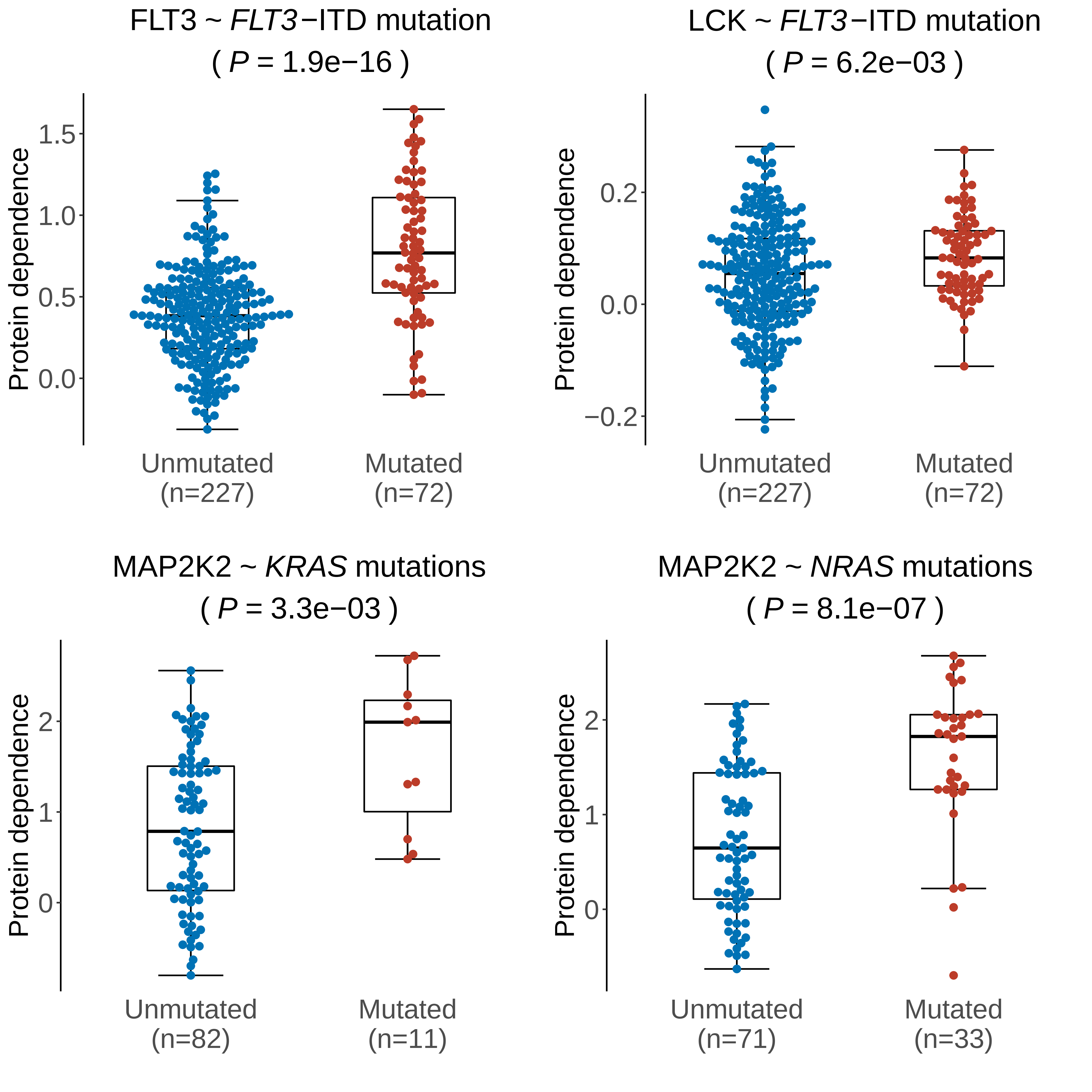

FLT3 ~ FLT3−ITD mutation
(P = 1.9e−16)
LCK ~ FLT3−ITD mutation
(P = 6.2e−03)
MAP2K2 ~ KRAS mutations
(P = 3.3e−03)
MAP2K2 ~ NRAS mutations
(P = 8.1e−07)
Protein dependence
1.5
1.0
0.5
0.0
0.2
0.0
−0.2
2
1
0
Unmutated
(n=227)
Mutated
(n=72)
Unmutated
(n=82)
Mutated
(n=11)
Unmutated
(n=71)
Mutated
(n=33)
